# A preliminary analysis of the association between perceived stigma and HIV-related pain in South Africans living with HIV

**DOI:** 10.4102/phcfm.v11i1.1647

**Published:** 2019-02-13

**Authors:** Antonia L. Wadley, Tamar Pincus, Michael Evangeli

**Affiliations:** 1 Brain Function Research Group, School of Physiology, University of the Witwatersrand, South Africa; 2 Department of Psychology, Royal Holloway University of London, United Kingdom

## Abstract

**Background:**

Stigma related to the human immunodeficiency virus (HIV) remains common and has been associated with severity of HIV-related symptoms. Associations between HIV stigma and HIV-related pain, one of the most common symptoms in HIV, have however not been investigated. Data from low back pain populations suggest that stigma is associated with worse pain intensity and so we hypothesised that the same would be the case in HIV.

**Aim:**

The goal of this study was to assess the association between HIV stigma and pain intensity in people living with HIV (PLWH) with chronic pain whilst controlling for depression, a well-established correlate of pain.

**Setting:**

The study took place at an HIV clinic in Johannesburg, South Africa.

**Methods:**

Mediation analysis was used to assess the effect of depression on the relationship between stigma and pain intensity in a cross-sectional cohort of 50 PLWH and chronic pain (pain most days of the week for > 3 months). All participants were assessed using the HIV/AIDS Stigma Instrument – PLWA, an 11-point numerical pain rating scale and the Beck Depression Inventory II.

**Results:**

In all, 88% (44/50) of participants reported experiencing some form of HIV stigma (HIV stigma scale score ≥ 1). Worst pain intensity and depressive symptoms individually correlated with total stigma score (Spearman’s *r* = 0.33, *p* = 0.02 for both). The mediation analysis highlighted that mediation of the relationship by depression was equivocal (*b* = -0.002, bootstrapped confidence interval -0.02 to 0.00).

**Conclusion:**

Whilst these preliminary data are marginal, they do suggest that associations between HIV stigma and HIV-related pain warrant further investigation. Future study should also include potential mechanisms, which may include mediation through depression.

## Introduction

Stigma towards people living with human immunodeficiency virus (HIV) remains prevalent. A survey of over 10 000 HIV-positive individuals in South Africa reported that over one-third perceived themselves as having experienced HIV stigma.^1^ Stigma against people living with HIV (PLWH) can take different forms including enacted stigma, which refers to acts perceived as stigmatising such as social exclusion or violence, or internalised stigma, whereby prevalent negative attitudes surrounding HIV are internalised and deemed valid by people living with HIV (PLWH).^2,3^

Greater perceived enacted stigma has been associated with increased HIV symptom severity.^4^ Associations between perceived HIV stigma and the experience of pain have not been investigated, however. Pain is one of the most common symptoms associated with HIV, affecting between 50% and 80% of PLWH.^5^ The pain frequently is of moderate to severe intensity and occurs concurrently at multiple body sites.^5^ About half of the pain is chronic (pain on most days for > 3 months).^6,7^

We recently reported that, in contrast to behaviours seen in many other chronic pain states, patients with chronic HIV-related pain reported not disclosing their pain to friends and family.^8^ The reason for this behaviour was that revealing their pain status may reveal their HIV status and the participants feared that this would lead to them being stigmatised.^8^ In individuals with chronic low back pain, stigma in the form of feeling guilty and unbelieved was associated with greater pain intensity and disability.^9^ Additionally, internalised stigma was associated with pain catastrophising (a cognitive style comprising frequent pain-related thoughts, rumination and feelings of helplessness)^10^ and reduced pain self-efficacy.^11^ Both increased pain catastrophising and reduced pain self-efficacy are associated with worse clinical outcomes, including increased pain intensity, in pain populations.^10,12^

These data lead us to hypothesise that HIV stigma is positively associated with pain intensity in PLWH with chronic pain. Moreover, given that HIV stigma is associated with greater depressive symptoms^3,13^ and depression is associated with greater pain intensity,^14^ the hypothesis here was that greater depressive symptoms would mediate the relationship between HIV stigma and pain intensity.

## Methods

### Study design

This was a cross-sectional study of patients visiting an HIV clinic in Johannesburg, South Africa, between April and June 2015.

### Setting

The clinic is a public, tertiary-care facility providing routine and specialist HIV-related care to PLWH in Johannesburg and surrounding areas. Data collection ran between April and June 2015.

### Study population and sampling strategy

Participants awaiting routine follow-up appointments with the clinic doctors were informed of the study and, if interested in finding out more, were taken to a private room. Participants willing to be recruited and who fulfilled the inclusion criteria provided written informed consent. Inclusion criteria were as follows: ≥18 years old, confirmed HIV-positive and the presence of pain most days of the week for at least the last 3 months. Two research assistants who were also fluent in local languages including isiZulu, isiXhosa and Sesotho assisted with consent and study procedures. The research assistants read the questionnaires to participants and clarified any unfamiliar terms.

Educational attainment was ascertained by asking participants which was the highest grade of schooling they had completed. It was then determined retrospectively whether they had completed primary, secondary or tertiary education. Participants’ employment status was determined by asking if they were currently employed full-time or part-time or if they were unemployed. Relevant clinical information regarding participants’ HIV history including time since diagnosis, time on antiretrovirals (ARVs), nadir CD4 T-cell count and viral load was retrieved from the participants’ medical files.

### Data collection

Worst pain intensity in the last week was measured on an 11-point numerical rating scale (NRS) anchored at 0 (‘no pain’) and 10 (‘worst pain imaginable’). This scale has good construct validity in a variety of populations^15,16^ including in African second-language English speakers.^17^

Depressive symptoms were measured using the Beck Depression Inventory (BDI) II,^18^ which is recommended for use in pain studies.^19^ The tool has 21 questions, which generate a score from zero to three for each question. The total score is thus 63, with a higher score indicating greater depressive symptoms. Scores between 13 and 19 indicate mild depression and a score ≥ 20 identifies moderate or severe depression.^20^ The BDI-II demonstrated good construct validity and internal consistency (α = 0.90) in an HIV-positive South African population.^21^ The internal consistency of the BDI-II was acceptable in this cohort (α = 0.78).

HIV-related stigma was measured using the HIV/AIDS Stigma Instrument – PLWA (HASI-P), which was developed and validated in five sub-Saharan African populations including a South African cohort.^22^ The tool has 33 questions, split into three sections. Section 1 includes 21 questions that assess events that have happened as a result of the participant’s HIV status being disclosed (e.g. ‘Someone mocked me when I passed by’). The second section contains seven questions related to stigma experienced in healthcare settings (e.g. ‘I was discharged from the hospital whilst still needing care’). The last section assesses participants’ thoughts and feelings on the disclosure of their HIV status (e.g. ‘I felt that I brought a lot of trouble to my family’). All statements are rated in terms of how often they have occurred in the last 3 months: ‘never’, ‘once or twice’, ‘several times’, ‘most of the time’. The sections can be further divided into six subsections: ‘verbal abuse’ (Q4, 9, 10, 11–13, 15, 19), ‘negative self-perception’ (Q29–Q33), ‘healthcare neglect’ (Q22–Q28), ‘social isolation’ (Q7, 8, 16–18), ‘fear of contagion’ (Q1–3, 5, 6, 14) and ‘workplace stigma’ (Q20–21). A mean of the scores for each subsection gives a score of zero to three for each subsection. Internal consistency of the subscales ranged from 0.76 to 0.91 and was 0.94 for the whole scale.^22^ As the subsections were highly correlated with the total stigma scale score of which they formed a part (see Table 1), we included the total score in the analyses as a proxy for the subsections. The internal consistency for the total score was found to be excellent in this cohort (α = 0.93).

**TABLE 1:** Pearson correlation coefficients between stigma subscales and total score.

Stigma subscale	Verbal abuse	Negative self-perception	Healthcare neglect	Social isolation	Fear of contagion	Workplace stigma	Stigma total
Verbal abuse	1.00	-	-	-	-	-	-
Negative self-perception	0.37	1.00	-	-	-	-	-
Healthcare neglect	0.07	−0.07	1.00	-	-	-	-
Social isolation	0.71	0.41	−0.02	1.00	-	-	-
Fear of contagion	0.48	0.44	−0.12	0.60	1.00	-	-
Workplace stigma	0.53	0.28	0.17	0.39	0.43	1.00	-
Stigma total	0.89	0.64	0.06	0.85	0.72	0.59	1.00

Participants were asked if they had disclosed their HIV status and, if so, whether to family, friends, work colleagues or their pastor. They were also asked, if they had not disclosed to someone, why they had chosen not to.

### Data analysis

Normally distributed variables were presented as mean (standard deviation) and non-normally distributed variables as median (range). Spearman’s correlations were used to correlate stigma with both worst pain intensity and BDI scores. The PROCESS tool^23^ was used to test the hypothesis that depression mediated the relationship between stigma score and worst pain intensity. Fifty participants were recruited, which was sufficient to detect a medium to large effect size with 0.8 power using the percentile bootstrap method.^24^ Statistical analyses were carried out using GraphPad Prism 6 (GraphPad, San Diego, CA, United States [US]), SPSS 2.15 (IBM, New York, US) and the PROCESS procedure for the mediational analysis. Finally, *p*-values ≤ 0.05 were considered significant.

### Ethical considerations

Ethical clearance was obtained from the Human Research Ethics Committee (Medical) of the University of the Witwatersrand (clearance no. M140877).

## Results

The cohort of 50 participants is described in Table 2. They were a city-dwelling, middle-aged, predominantly female cohort and had a high level of unemployment. All participants were on stable antiretroviral therapy. Worst pain experienced in the last week was generally severe (7–10 on NRS): the median pain intensity was 8/10, with 62% (31/50) experiencing pain intensity at 8/10 or greater. The majority had more than one pain site, with head and spine being the most frequent sites of pain (both: 40%, 20/50). Forty-eight per cent (24/50) scored > 20 on the BDI-II (moderate or severe depression) and 24% (12/50) scored between 13 and 19 (mild depression).

**TABLE 2:** Demographic characteristics of the cohort.

Variable	Value
Age (years)^†^	45 (10)
Gender – Female	44 (88)
Highest level of education^‡,§^	
No education	6 (12)
Primary	16 (32)
Secondary	23 (46)
Tertiary	4 (8)
Employment status^§^	
Full-time	21 (42)
Part-time or piecework	7 (14)
Unemployed	22 (44)
Years since diagnosis^†^	8 (5)
Years on antiretroviral therapy^†^	5 (3)
Nadir CD4 T-cell count (cells.mm^−3^)^¶^	152 (2–426)
Number with undetectable viral load (< 40 copies/ml)^†^	39 (78)
Worst pain intensity (0–10)^¶^	8 (4–10)
Number of pain sites^¶^	2 (1–4)
Beck Depression Inventory (0–63)^†^	19 (8)
Total stigma score (0–99)^¶^	7 (0–64)

*n* = 50.

† , Mean (SD).

‡ , Missing data for one participant.

§ , Count (%).

¶ , Median (range).

Overall, 88% (44/50) participants reported experiencing some form of HIV stigma (stigma scale score ≥ 1) in the preceding 3 months. Anecdotally, whilst completing the stigma tool, participants frequently reported that the enacted stigma statements were not relevant to them because they had not disclosed their HIV status. Indeed, whilst 98% (49/50) of participants had disclosed their status to someone they trusted, 62% (31/50) of patients reported that they had specifically not disclosed their HIV status to their remaining family, friends or co-workers because they feared being stigmatised.

### Univariate analysis

The intensity of worst pain in the last week correlated with total stigma score (Spearman’s *r* = 0.33; *p* = 0.02). Similarly, greater depressive symptoms (BDI score) correlated with total stigma score (Spearman’s *r* = 0.33; *p* = 0.02).

### Mediation analysis

Mediation analysis was used to determine whether depression mediated the effect of stigma on worst pain intensity. The direct effect of stigma on worst pain intensity was on the threshold of significance in this model (*b* = 0.03, confidence interval [CI] 0.00 to 0.07, *p* = 0.05). The indirect effect of stigma on worst pain intensity through depression was tested using a bootstrap estimation (*n* = 1000). The upper limit of the CI was zero, making the interpretation of whether depression mediated the relationship between total stigma and worst pain intensity equivocal (*b* = -0.002, bootstrapped CI -0.02 to 0.00).

## Discussion

The hypothesis for the study was that HIV stigma would be associated with pain intensity and that depressive symptoms would mediate the relationship. The results showed that HIV stigma was associated with worst pain intensity, but whether this relationship was mediated by depression is unclear.

Perceived HIV stigma was found to be highly prevalent in this cohort (88% of participants), which is far greater than the prevalence of enacted (~30%) and internalised (35%) stigma reported in a general survey of South Africans living with HIV.^1^ However, because only patients with pain were recruited, it is not clear if HIV stigma is associated with risk of having pain, but this would be a useful objective of future studies.

Contrary to our hypothesis, the relationship between stigma and worst pain intensity was not mediated by depression. Future work needs to determine which other psychosocial factors might mediate the relationship between stigma and worst pain intensity. In an Australian chronic pain cohort, pain catastrophising was associated with internalised stigma,^11^ and a systematic review of psychological symptoms in people with HIV on treatment identified higher levels of anxiety than other populations with chronic health issues.^25^ Stigma causes social withdrawal and exclusion^11^ and, in an experimental setting, mild to moderate social exclusion led to increased pain sensitivity.^26^ Thus, pain catastrophising, anxiety and social support may be psychosocial factors worth including in future studies of HIV stigma and pain. Additionally, recent work suggested that PLWH and chronic pain may maintain their activity levels despite high pain intensity in order to avoid HIV stigma.^8^ Thus, physical activity could also be investigated as a mediator of the association between perceived HIV stigma and pain intensity.

The burden of pain and depression in this cohort was high. For example, the median pain intensity was 8/10 with over half of the sample experiencing pain intensity of 8/10 or greater. Additionally, almost 50% of the sample scored moderate or severe on the depression scale. Whilst the mean BDI score for this cohort (19; SD 8) was greater than that reported by healthy controls (9; SD 8), it was similar to another cohort of HIV-positive South Africans (17; SD 12).^21,27^ It is worth considering that individuals living with both HIV and chronic pain live with two stigmatised conditions and this may have compounded both their depressive symptoms and pain intensity,^28^ leading to the high scores seen here.

There were limitations to the study. It is possible that the sensitivity of the questionnaires may have been reduced by non-first-language English speakers answering English-language questionnaires. This was a cross-sectional study and so it cannot be confirmed that stigma leads to pain. Indeed, the reverse is possible, where worse pain intensity could lead to greater perceived stigma. Similarly, people with more pain may experience greater depression and consequently be at risk for greater experiences of stigma. Longitudinal studies are required to explore the relationship between pain and HIV stigma further. Indeed, data from other longitudinal studies suggest worse physical and mental health (both of which may indicate greater presence and intensity of pain) predicted increases in internalised stigma.^29^ We recruited patients from an HIV clinic at a tertiary hospital that, in addition to routine care, specialises in dealing with patients failing routine antiretroviral therapy. Thus, some patients included in this study may have had worse mental and physical health than a general population of PLWH and, by this logic, may have been at greater risk of HIV stigma. Our cohort was approximately 90% female. Typically, we recruit 70% women in studies in similar Johannesburg cohorts and so men were under-represented here. The generalisability of our findings may be limited, therefore. The stigma scale we used, the HASI-P, included primarily, the construct of enacted stigma and a single subscale on internalised stigma.^2^ Fear of anticipated stigma, the anticipation of future experiences of enacted stigma,^2^ was highlighted by the qualitative answers, suggesting that a proportion of participants had not disclosed their HIV status for fear of enacted stigma. Thus, anticipated stigma would be an important factor to assess in future work. Furthermore, it would be beneficial to include a more robust measurement of internalised stigma to determine the impact of enacted stigma and also perceived stigma within the community.^30^ Determining the association between each of these subtypes of HIV stigma and HIV-related pain would be an interesting aim of future work. Another limitation was our small sample size. This study was conducted as a pilot study to test our hypothesis that HIV stigma would be associated with intensity of HIV-related pain. Following the mediation analysis, the CIs generated from the association between stigma and pain intensity identified that the effect might be marginal, which highlights that the association needs to be repeated. We mentioned the high scores for pain ratings and depressive symptoms. It is possible that ceiling effects for the pain rating scale and BDI may have reduced our ability to elucidate significant mediation. These findings of high pain and depression are typical of this poor, urban cohort, however,^31^ and may be difficult to remedy in future.

## Conclusion

In this first assessment of HIV-related pain and stigma, we found an association between perceived HIV stigma and increased pain intensity. The role of depression in mediating this relationship was not clear. Further study is warranted to repeat these findings and determine the mechanisms by which stigma may influence HIV-related pain, including the role of depression. This information is important to inform psychosocial interventions for HIV-related pain. For example, a recent programme piloted in the US for PLWH, pain and depressive symptoms^32^ may be more effective if perceived stigma is also addressed. Furthermore, behavioural interventions for managing pain in HIV^33^ may need to take into account that stigma may be a barrier to functional coping with pain.

## References

[1] Human Research Council The people living with HIV stigma index: South Africa 2014 2015 [homepage on the Internet]. [cited 2016 May 18]. Available from: http://www.stigmaindex.org/sites/default/files/reports/Summary-Booklet-on-Stigma-Index-Survey%20South%20Africa.pdf

[2] Earnshaw VA, Chaudoir SR From conceptualizing to measuring HIV stigma: A review of HIV stigma mechanism measures. AIDS Behav. 2009;13(6):1160–1177. PubMed PMID: Pubmed Central PMCID: 10.1007/s10461-009-9593-3 19636699 PMC4511707

[3] Steward WT, Herek GM, Ramakrishna J, et al HIV-related stigma: Adapting a theoretical framework for use in India. Soc Sci Med. 2008;67(8):1225–1235. PubMed PMID: Pubmed Central PMCID: 10.1016/j.socscimed.2008.05.032 18599171 PMC2603621

[4] Earnshaw VA, Smith LR, Chaudoir SR, Amico KR, Copenhaver MM HIV stigma mechanisms and well-being among PLWH: A test of the HIV stigma framework. AIDS Behav. 2013;17(5):1785–1795. PubMed PMID: Pubmed Central PMCID: 10.1007/s10461-013-0437-9 23456594 PMC3664141

[5] Parker R, Stein DJ, Jelsma J Pain in people living with HIV/AIDS: A systematic review. J Int AIDS Soc. 2014;17:18719. PubMed PMID: Pubmed Central PMCID: 10.7448/IAS.17.1.18719 24560338 PMC3929991

[6] Merskey H, Bogduk N, editors Classification of chronic pain: Descriptions of chronic pain syndromes and definitions of pain terms. 2nd ed. Seattle, DC: IASP Press; 2002.

[7] Mphahlele NR, Kamerman PR, Mitchell D Progression of pain in ambulatory HIV-positive South Africans. Pain Manag Nurs. 2015;16(1):e1–e8. PubMed PMID: 10.1016/j.pmn.2014.05.013 25175556

[8] Wadley AL, Mitchell D, Kamerman PR Resilience does not explain the dissociation between chronic pain and physical activity in HIV-positive South Africans. PeerJ. 2016;4:e2464 10.7717/peerj.2464 27672513 PMC5028784

[9] Serbic D, Pincus T Pain-related guilt in low back pain. Clin J Pain. 2014;30(12):1062–1069. PubMed PMID: 10.1097/AJP.0000000000000059 25329140

[10] Quartana PJ, Campbell CM, Edwards RR Pain catastrophizing: A critical review. Expert review of neurotherapeutics. 2009;9(5):745–758. PubMed PMID: Pubmed Central PMCID: 10.1586/ern.09.34 19402782 PMC2696024

[11] Waugh OC, Byrne DG, Nicholas MK Internalized stigma in people living with chronic pain. J Pain. 2014;15(5):550 e1–e10. PubMed PMID: . 24548852 10.1016/j.jpain.2014.02.001

[12] Jackson T, Wang Y, Wang Y, Fan H Self-efficacy and chronic pain outcomes: A meta-analytic review. J Pain. 2014;15(8):800–814. PubMed PMID: 10.1016/j.jpain.2014.05.002 24878675

[13] Lee RS, Kochman A, Sikkema KJ Internalized stigma among people living with HIV-AIDS. AIDS Behav. 2002;6(4):309–319. 10.1023/A:1021144511957

[14] Glombiewski JA, Hartwich-Tersek J, Rief W Depression in chronic back pain patients: Prediction of pain intensity and pain disability in cognitive-behavioral treatment. Psychosomatics. 2010;51(2):130–136. PubMed PMID: 10.1016/S0033-3182(10)70673-3 20332288

[15] Jensen MP, Karoly P, Braver S The measurement of clinical pain intensity: A comparison of six methods. Pain. 1986;27(1):117–126. PubMed PMID: 10.1016/0304-3959(86)90228-9 3785962

[16] Ruskin D, Lalloo C, Amaria K, et al Assessing pain intensity in children with chronic pain: Convergent and discriminant validity of the 0 to 10 numerical rating scale in clinical practice. Pain Res Manag. 2014;19(3):141–148. PubMed PMID: Pubmed Central PMCID: 10.1155/2014/856513 24712019 PMC4158959

[17] Mphahlele N, Mitchell D, Kamerman P Validation of the Wisconsin Brief Pain Questionnaire in a multilingual South African population. J Pain Symptom Manag. 2008;36(4):396–412. PubMed PMID: eng. 10.1016/j.jpainsymman.2007.10.020 18448308

[18] Beck AT, Steer RA, Brown GK Manual for the Beck depression inventory. 2nd ed. San Antonio, TX: The Psychological Corporation; 1996.

[19] Dworkin RH, Turk DC, Wyrwich KW, et al Interpreting the clinical importance of treatment outcomes in chronic pain clinical trials: IMMPACT recommendations. J Pain. 2008;9(2):105–121. PubMed PMID: 10.1016/j.jpain.2007.09.005 18055266

[20] Dozois DJA, Dobson KS, Ahnberg JL A psychometric evaluation of the Beck Depression Inventory-II. Psychol Assess. 1998;10(2):83–89. 10.1037/1040-3590.10.2.83

[21] Kagee A, Nel A, Saal W Factor structure of the Beck depression inventory-II among South Africans receiving antiretroviral therapy. AIDS care. 2014;26(2):257–262. PubMed PMID: 10.1080/09540121.2013.802278 23750786

[22] Holzemer WL, Uys LR, Chirwa ML, et al Validation of the HIV/AIDS stigma instrument – PLWA (HASI-P). AIDS care. 2007;19(8):1002–1012. PubMed PMID: 10.1080/09540120701245999 17851997

[23] Hayes AF Introduction to mediation, moderation and conditional process analysis. New York: Guilford Press; 2013.

[24] Fritz MS, Mackinnon DP Required sample size to detect the mediated effect. Psychol Sci. 2007;18(3):233–239. PubMed PMID: Pubmed Central PMCID: 10.1111/j.1467-9280.2007.01882.x 17444920 PMC2843527

[25] Lowther K, Selman L, Harding R, Higginson IJ Experience of persistent psychological symptoms and perceived stigma among people with HIV on antiretroviral therapy (ART): A systematic review. Int J Nurs Stud. 2014;51(8):1171–1189. PubMed PMID: 10.1016/j.ijnurstu.2014.01.015 24602830

[26] Bernstein MJ, Claypool HM Social exclusion and pain sensitivity: Why exclusion sometimes hurts and sometimes numbs. Pers Soc Psychol Bull. 2012;38(2):185–196. PubMed PMID: 10.1177/0146167211422449 21885860

[27] Whisman MA, Richardson ED Normative data on the Beck depression inventory-Second edition (BDI-II) in college students. J Clin Psychol. 2015;71(9):898–907. PubMed PMID: . 25950150 10.1002/jclp.22188

[28] Goodin BR, Owens MA, White DM, et al Intersectional health-related stigma in persons living with HIV and chronic pain: Implications for depressive symptoms. AIDS care. 2018;30(2):66–73. PubMed PMID: Pubmed Central PMCID: 10.1080/09540121.2018.1468012 29848042 PMC6080251

[29] Pantelic M, Shenderovich Y, Cluver L, Boyes M Predictors of internalised HIV-related stigma: A systematic review of studies in sub-Saharan Africa. Health Psychol Rev. 2015;9(4):469–490. PubMed PMID: 10.1080/17437199.2014.996243 25559431

[30] Turan B, Budhwani H, Fazeli PL, et al How does stigma affect people living with HIV? The mediating roles of internalized and anticipated HIV stigma in the effects of perceived community stigma on health and psychosocial outcomes. AIDS Behav. 2017;21(1):283–291. PubMed PMID: Pubmed Central PMCID: 10.1007/s10461-016-1451-5 27272742 PMC5143223

[31] Pillay P, Wadley AL, Cherry CL, Karstaedt AS, Kamerman PR Psychological factors associated with painful versus non-painful HIV-associated sensory neuropathy. AIDS Behav. 2018;22:1584–1595. PubMed PMID: 10.1007/s10461-017-1856-9 28710709

[32] Uebelacker LA, Weis berg RB, Herman DS, et al Pilot randomized trial of collaborative behavioral treatment for chronic pain and depression in persons living with HIV/AIDS. AIDS Behav. 2016;20(8):1675–1681. PubMed PMID: Pubmed Central PMCID: 10.1007/s10461-016-1397-7 27115400 PMC4945374

[33] Parker R, Jelsma J, Stein DJ Managing pain in women living with HIV/AIDS: A randomized controlled trial testing the effect of a six-week peer-led exercise and education intervention. J Nerv Ment Dis. 2016;204(9):665–672. PubMed PMID: 10.1097/NMD.0000000000000506 27002748

